# Associations of non-motor symptoms with perceptual speech impairments in Parkinson’s disease

**DOI:** 10.3389/fneur.2026.1827374

**Published:** 2026-06-24

**Authors:** Rui Liu, Ciara Leydon

**Affiliations:** 1 Department of Health Sciences, Sacred Heart University, Fairfield, CT, United States; 2 Department of Communication Disorders, Sacred Heart University, Fairfield, CT, United States

**Keywords:** non-motor symptoms, Parkinson’s disease, perceived speech impairment, PPMI (Parkinson’s Progression Markers Initiative), sex-differences

## Abstract

**Background:**

Communication impairments are common, multifaceted, and progressive symptoms of Parkinson’s disease (PD), and have deleterious impacts on individual wellbeing. Self-awareness of communication impairments and the rate and extent of decline in speech skills vary substantially across individuals and are challenging to predict. To address these, we aimed to (1) evaluate associations between non-motor symptoms (NMS) and speech impairment at year seven and (2) compare the progression of self-perceived and examiner-rated speech impairment ratings scores across a seven-year follow-up period.

**Method:**

The study included 417 PD participants from the Parkinson’s Progression Marker Initiative. NMS were assessed using validated instruments covering sleep, olfaction, neurobehavioral, autonomic, and neuropsychological domains. Speech severity was measured using the Movement Disorder Society–Unified Parkinson’s Disease Rating Scale. Multivariate ordinal logistic regression examined associations between baseline NMS and speech severity at year seven. Changes in speech severity scores over seven years were analyzed using Friedman’s test.

**Results:**

Several NMS were associated with self-perceived and examiner-rated speech severity at year seven. Baseline REM sleep behavior disorder and autonomic dysfunction were associated with significantly higher odds of speech impairment at year seven. For self-perceived speech, odds ratios (OR) and 95% confidence interval (CI) were 1.58 (1.18–2.12) for REM sleep behavior disorder and 1.71 (1.24–2.36) for autonomic dysfunction. For examiner-rated speech, ORs were 1.45 (1.06–1.98) and 1.71 (1.21–2.42), respectively. Daytime sleepiness was associated with worse examiner-rated speech scores at year seven (OR = 1.51, 95% CI 1.10–2.09). Both self-perceived and examiner-rated speech impairments worsened over seven years (*p* < 0.001), with no significant differences between the two assessments (*p* > 0.05).

**Conclusion:**

Several NMS were associated with worse speech ratings at year seven. These simple and readily accessible instruments may help identify individuals at greater risk of worsening speech skills, with the goal of enabling timely and targeted speech and language therapy. The consistency between self-perceived and examiner-rated speech scores suggests individuals with PD maintain good insight into their speech impairments, despite a decline in communication abilities.

## 1 Introduction

Parkinson’s disease (PD) is a common neurogenerative disorder characterized by progressive loss of dopaminergic neurons in the substantia nigra pars compacta and is associated with a broad range of motor and non-motor symptoms (NMS) (1). One prevalent and challenging motor symptom is a decline in communication abilities, including speech skills (2). At initial diagnosis, 40% of people with Parkinson’s disease (PWPD) exhibit speech impairments (3), typically hypokinetic dysarthria, characterized by impaired vocal quality, monopitch, monoloudness, and imprecise articulation causing poor speech intelligibility (4). As the disease progresses, up to 90% of PWPD experience speech impairments (5), with varying symptoms and phenotypes (6). Given that speech is a multi-system complex sensorimotor process involving rapid, precise, coordinated respiratory, laryngeal, and vocal tract movements, multiple factors may contribute to worsening speech skills. Demographic factors, including age of onset and sex, have been associated with differential progression of motor symptoms and speech deficits (7, 8). Recent studies have also reported a link between PD motor phenotype and both self-perceived and instrumental measures of speech (9). However, predictors of a decline in speech remain elusive.

While motor symptoms of rigidity, bradykinesia, and tremor are hallmarks of a clinical diagnosis of PD, by the time these symptoms occur, extensive loss of nigrostriatal neurons has occurred (1). Certain NMS, such as olfactory impairment and rapid eye movement sleep behavior disorder (RBD), may reflect these early neuropathological, preclinical changes (1). Longitudinal epidemiologic studies have consistently shown that some NMS occur years, or even decades, before PD diagnosis and have been investigated as potential markers of prodromal PD (10). For example, about 40% of PWPD present with a mild cognitive impairment at diagnosis of PD (11, 12), with prevalence of cognitive impairment increasing over time. Similarly, depression can disrupt the rhythm of speech resulting in a monotone pitch or reduced loudness (13), exacerbating the symptoms commonly associated with PD-related hypokinetic dysarthria. Other NMS, such as autonomic dysfunction and sleep behaviors have been reported to predict severity of examiner ratings of PD-related speech symptoms (3). Since multiple NMS can be readily assessed using simple screeners or tests, it warrants consideration whether they could serve as potential predictors of speech impairment in PD.

Accurate self-perception of changes in speech skills is crucial for adjusting speech behaviors to achieve communicative success. Although PWPD frequently report speech difficulties, subjective assessment of their speech may differ in severity from that identified through clinical evaluation (14). While longitudinal studies have reported worsening impairments in examiner-rated speech (15, 16), few examined longitudinal changes in self-perceived speech function (9). Further, only one study compared self-perceived and examiner-rated speech in PWPD. Pawlukowska et al. (17) found that, while some correlation exists between subjective perception of speech severity and objective measures, PDWP did not perceive their speech disorders as having a significant impact on their quality of speech. This study, however, did not examine how self-perceived and objectively measured speech differs over time (17). Comparing alignment between self-perceived and examiner-rated speech deficits over time could enhance our understanding of the evolution of speakers’ awareness of their functional speech impairments as speech symptoms progress. Given the paucity of existing research, the objectives of the current study were twofold: (1) to examine associations between baseline NMS and speech ratings at year seven, and whether these associations vary by sex, and (2) to compare the progression of self-perceived and examiner-rated speech impairment ratings scores across a seven-year follow-up period. Insights from the current study could help identify individuals at greater risk for speech impairments, with the ultimate goal of enabling timely and targeted speech and language therapeutic services.

## 2 Materials and methods

### 2.1 Data source and study participants

We used data from the Parkinson’s Progression and Markers Initiative (PPMI), a longitudinal multicenter study that collects comprehensive demographic and clinical data aimed to explore markers of PD risk, onset and progression. Detailed PPMI protocol and methodology have been described elsewhere (18).^1^ Initial recruitment of the PPMI cohort began in 2010. During the initial enrollment phase, 423 newly diagnosed drug-naïve PD patients met study inclusion criteria (18) and underwent comprehensive assessments. Using the analytic cohort classification defined by the PPMI steering committee and excluding participants labeled ‘not-PD’, data for 417 PD participants aged 30 years and older (average age 61.6 ± 9.8 years) were obtained for our study. Of those, 174 (42%) PD participants (121 male and 53 female) had complete speech assessments data from baseline through year 7 (“completers”). The remaining 243 participants (58%) were referred to as “non-completers” in the following analysis. All data for the current study were downloaded from the PPMI database^2^ according to guidelines. Each participating PPMI site obtained written informed consent from all participants and received approval from an ethical standards committee. The data used in this study were fully de-identified and publicly available, and the study was exempted from ethical approval by the Sacred Heart University Institutional Review Board (IRB-FY2025-315).

### 2.2 Demographics and clinical assessments

Demographic evaluations and comprehensive clinical examinations were performed by investigators at individual study sites. Motor functions were evaluated using the Movement Disorder Society-sponsored revision of the Unified Parkinson’s Disease Rating Scale (MDS-UPDRS) Part III-motor ratings. Hoehn and Yahr staging was used to determine PD symptom progression and disease severity. For subtype stratification, we classified PD phenotype into tremor dominant, postural instability and gait disturbance, or intermediate-PD using previously published methods (19).

### 2.3 NMS assessments

NMS assessments at baseline included an extensive set of validated tests and questionnaires that examined five major areas of non-motor functions: sleep, olfaction, neurobehavioral, autonomic function, and cognition (Supplementary Table S1). Sleep disturbance was evaluated by the Epworth Sleepiness Scale (ESS) and RBD Screening Questionnaire (RBDSQ). Olfaction was assessed by the University of Pennsylvania Smell Identification Test (UPSIT). Neurobehavioral assessments included the State–Trait Anxiety Inventory (STAI) and the 15-item version of the Geriatric Depression Scale (GDS-15). Autonomic dysfunction was evaluated using the Scales for Outcomes in Parkinson’s disease – Autonomic (SCOPA-AUT). Cognition was evaluated with Montreal Cognitive Assessment (MoCA) as a global cognitive screening test, and a battery of validated neuropsychological tests evaluating four major cognitive domains: memory, visuospatial functions, working memory-executive functions, and attention-processing speed. A composite score was created for each cognitive domain with higher scores indicating better cognitive performance (Supplementary Table S1).

### 2.4 Speech assessments

Speech assessment ratings were obtained using MDS-UPDRS Part-II.1 and Part III.1. The MDS-UPDRS Part II.1 elicited participant ratings of self-perceived speech difficulty over the past week on a 5-point scale ranging from 0–4, with zero indicating normal speech and four indicating severe speech impairment. In MDS-UPDRS Part III.1, examiners rated participants’ speech and conversation abilities, also using a five-point scale.

### 2.5 Statistical analysis

We presented means and standard deviations for continuous variables and proportions for categorical variables. To assess differences between groups, we performed the Mann–Whitney U test for continuous demographic and clinical variables and Chi-square test for categorical variables. We examined potential associations between NMS and self-perceived and examiner-rated speech impairment ratings at year seven using multivariate ordinal logistic regression models. All NMS variables were standardized to z-scores to make the *β* coefficient and odds ratios directly comparable across symptoms. All models were adjusted for age, sex, race, education, disease duration, UPDRS III motor scores, and H&Y stage. We conducted subgroup analyses by sex. Longitudinal changes in self-perceived and examiner-rated speech severity scores across baseline and seven subsequent visits were analyzed using Friedman’s repeated measures analysis of variance (ANOVA) tests. We further conducted Wilcoxon signed-rank tests with Bonferroni correction to compare differences between self-perceived and examiner-rated speech ratings at each visit over the 7-year follow-up. To examine whether longitudinal speech trajectories differed by sex, ordinal generalized estimating equation (GEE) models were conducted separately for repeated measurements of self-perceived and examiner-rated speech severity scores collected across follow-up visits. Statistical analyses were carried out using SAS, version 9.4 (SAS Institute Inc., Cary, NC) and IBM SPSS Statistics 29 (IBM Corp, Armonk, NY) and. All statistical tests were 2-sided with a significance level of with *α* = 0.05.

## 3 Results

### 3.1 Demographic characteristics

Baseline demographics and clinical characteristics were similar between completers and non-completers, except for disease severity. A significantly higher proportion of non-completers (61.3%) were in stage 2 of H&Y compared to completers (47.7%), *p* = 0.02. Demographic and general clinical characteristics of PD cases are summarized in Table 1 by sex. Male and female patients were comparable in baseline demographics and most clinical motor presentations, including self-perceived and examiner-rated speech ratings. However, among completers, male cases were more likely to have reported bradykinesia at diagnosis (*p* = 0.03). Baseline global cognition was significantly lower among male patients compared to female patients (*p* = 0.005).

**Table 1:** Demographic and clinical characteristics of PD patients at enrollment, by sex.

Characteristics	Enrolled (*N* = 417)			Completers^a^ (*N* = 174)		
	Male (*N* = 273)	Female (*N* = 144)	*p* value^b^	Male (*N* = 121)	Female (*N* = 53)	*p* value^b^
Age at enrollment, y	62.1 (9.8)	60.7 (9.7)	0.08	61.3 (10.0)	59.0 (9.2)	0.09
Race, % white	96.0	94.4	0.48	98.4	96.2	0.39
Education, y	15.7 (2.9)	15.3 (3.1)	0.11	15.7 (3.0)	15.1 (2.7)	0.08
Disease duration, mo	6.2 (5.9)	7.1 (7.5)	0.49	6.4 (6.2)	6.6 (6.8)	0.66
Global cognition	26.9 (2.3)	27.6 (2.2)	**0.004**	26.8 (2.3)	27.6 (2.5)	**0.005**
Tremor at diagnosis, %	78.0	77.8	0.95	81.0	77.4	0.58
Rigidity at diagnosis, %	78.8	70.1	0.14	82.6	69.8	0.10
Bradykinesia at diagnosis, %	83.2	80.6	0.79	88.4	75.5	**0.03**
Postural instability at diagnosis, %	7.0	6.9	0.34	5.0	9.4	0.35
UPDRS Part III score	21.3 (9.1)	20.2 (8.4)	0.31	20.4 (8.9)	19.3 (8.2)	0.50
Hoehn and Yahr stage, median	2.0	2.0	0.59	2.0	1.0	0.46
PD motor phenotype, %						
TD	72.5	69.4	0.71	71.1	67.9	0.58
PIGD	16.9	20.1		16.5	22.6	
IND	10.6	10.4		12.4	9.4	

IND = intermediate; PD = Parkinson disease; PIGD = postural instability and gait disturbance; TD = tremor dominant; UPDRS = Unified Parkinson’s Disease Rating Scale. Data are given as mean (SD) for continuous variables, and percentage (%) for categorical variables. Bold text indicates statistically significant (*p* < 0.05).

a Completers are those among the enrolled cases who completed all annual speech assessments from baseline to year 7.

b Based on Mann–Whitney U test or Chi-square test.

### 3.2 Associations of baseline NMS with self-perceived and examiner-rated speech severity

Associations between baseline demographic, motor, and non-motor factors with self- and examiner-rated speech impairment at year seven are shown in Table 2. Males had significantly higher odds of experiencing greater self-perceived (odds ratio [OR] = 2.12; 95% confidence interval [CI]: 1.13–3.97) and examiner-rated (OR = 3.43; 95% CI: 1.74–6.80) speech impairment severity ratings at year seven. Several NMS measured at baseline were significantly associated with speech ratings in year seven (Table 2). Each one-unit increase in baseline RBDSQ z-score was significantly associated with higher odds of severe self-perceived (OR = 1.58, 95% CI: 1.18–2.12) and examiner-rated (OR = 1.45, 95% CI: 1.06–1.98) speech impairment ratings. A unit increase in SCOPA-AUT was also associated with significantly higher self-perceived (OR = 1.71, 95% CI: 1.24–2.36) and examiner-rated (OR = 1.71, 95% CI: 1.21–2.42) speech impairment ratings. Additionally, higher baseline daytime sleepiness was strongly associated with higher examiner-rated speech severity rating at year seven (OR = 1.51, 95% CI: 1.10–2.09), but not with self-perceived speech severity. Baseline motor phenotype was not significantly associated with self-perceived or examiner-rated speech severity at year seven. Sensitivity analyses were conducted using delta scores from baseline to year 7 as the outcome to assess changes in speech severity over follow-up (Supplementary Table S2). Overall, the direction of associations was largely consistent with those observed in the primary analyses. Notably, higher baseline RBDSQ scores were associated with greater worsening in self-perceived speech severity over follow-up (*β* = 0.19, 95% CI: 0.04–0.34), but not with examiner-rated speech severity. No significant associations were observed between SCOPA-AUT and change in speech severity over follow-up.

**Table 2:** Associations of baseline demographic, NMS, and motor subtypes with speech severity rating at year 7.

Baseline variables	Self-perceived speech severity rating in year 7			Examiner-rated speech severity rating in year 7		
	OR	95% CI	*p* value^a^	OR	95% CI	*p* value^a^
Baseline demographics						
Age	1.03	0.99–1.06	0.07	1.03	0.99–1.06	0.08
Sex (male vs. female)	2.12	1.13–3.97	**0.02**	3.43	1.74–6.80	**0.0004**
Race (white vs. other)	0.28	0.04–1.73	0.17	0.13	0.02–0.92	**0.04**
Education	1.02	0.92–1.12	0.76	0.88	0.79–0.97	**0.01**
Disease duration	0.97	0.92–1.01	0.13	1.02	0.97–1.07	0.49
UPDRS motor score	1.02	0.98–1.06	0.32	1.04	0.99–1.08	0.10
H&Y stage	1.38	0.60–2.16	0.69	1.53	0.77–3.07	0.23
Sleep disorder						
Epworth sleepiness scale	1.27	0.95–1.71	0.11	1.51	1.10–2.09	**0.01**
RBDSQ	1.58	1.18–2.12	**0.002**	1.45	1.06–1.98	**0.02**
Olfactory						
UPSIT	0.94	0.67–1.33	0.74	1.42	0.97–2.07	0.07
Neurobehavioral						
Total anxiety	1.02	0.77–1.36	0.88	1.03	0.76–1.41	0.84
State anxiety	0.94	0.71–1.23	0.64	0.96	0.71–1.29	0.78
Trait anxiety	1.14	0.85–1.52	0.39	1.12	0.82–1.53	0.46
Geriatric depression	0.93	0.72–1.20	0.57	0.99	0.73–1.35	0.95
Cognitive domains^b^						
Global	0.93	0.72–1.20	0.57	0.96	0.73–1.27	0.79
Memory	0.91	0.58–1.43	0.67	0.69	0.42–1.13	0.14
Visuospatial	0.96	0.70–1.32	0.80	0.82	0.58–1.15	0.25
Working memory-executive	1.04	0.78–1.40	0.82	1.05	0.76–1.45	0.76
Attention-processing speed	0.83	0.60–1.16	0.28	0.72	0.50–1.03	0.07
Autonomic						
SCOPA-AUT	1.71	1.24–2.36	**0.001**	1.71	1.21–2.42	**0.003**
PD motor subtypes						
PIGD vs. TD	1.29	0.62–2.66	0.50	0.85	0.39–1.87	0.68
IND vs. TD	1.05	0.42–2.61	0.91	0.98	0.37–2.62	0.96

CI = Confidence Interval; OR = Odds Ratio; IND = intermediate; PD = Parkinson disease; PIGD = postural instability and gait disturbance; TD = tremor dominant; UPDRS = Unified Parkinson’s Disease Rating Scale. Bold text indicates statistically significant (*p* < 0.05).

a Adjusted for age at baseline, race, education, disease duration, UPDRS III motor scores, and H&Y stage.

b The global domain included the Montreal cognitive assessment test. The memory domain included the immediate recall, delayed recall, and delayed recognition of the Hopkins verbal learning test–revised. The visuospatial domain included the Benton judgment of line orientation. The working memory–executive domain included the letter number sequencing and the semantic fluency–animal tests. The attention processing speed included the symbol digit modalities test.

### 3.3 Sex-specific associations of baseline NMS with self-perceived and examiner-rated speech severity

To examine potential sex-related differences in the association between baseline NMS and speech severity ratings at year seven, we further conducted stratified analyses by sex (Table 3). For females with PD, each one-unit increase in Epworth Sleepiness Scale (OR = 1.78, 95% CI: 1.00–3.18) and SCOPA-AUT (OR = 2.05, 95% CI: 1.12–3.74) z-scores were associated with higher odds of more severe self-perceived speech ratings. In males with PD, higher baseline RBDSQ z-score was significantly associated with higher self-perceived speech severity rating (OR = 1.51, 95% CI: 1.07–2.12). For examiner-rated speech ratings, higher baseline RBDSQ z-score (OR = 2.16, 95% CI: 0.99–4.73) and UPSIT (OR: 2.37, 95% CI: 1.11–5.05) were associated with higher odds of severe ratings in female PD patients. In contrast, higher baseline SCOPA-AUT z-scores were associated with higher odds severe examiner-rated speech ratings (OR = 1.75, 95% CI: 1.12–2.73) in male PD patients.

**Table 3:** Associations of NMS and motor subtypes with speech severity ratings at year 7, by sex.

NMS and PD motor subtypes	Self-perceived speech severity rating in year 7						Examiner-rated speech severity rating in year 7						Male (*N* = 121)			Female (*N* = 53)			Male (*N* = 121)			Female (*N* = 53)			OR	95% CI	*p* value^a^	OR	95% CI	*p* value^a^	OR	95% CI	*p* value^a^	OR	95% CI	*p* value^a^
Sleep disorder												
Epworth sleepiness scale	1.05	0.72–1.54	0.793	1.78	1.00–3.18	**0.05**	1.35	0.89–2.06	0.16	1.80	0.98–3.32	0.06
RBDSQ	1.51	1.07–2.12	**0.02**	1.40	0.69–2.83	0.35	1.22	0.84–1.75	0.29	2.16	0.99–4.73	**0.05**
Olfactory												
UPSIT	0.78	0.52–1.18	0.2	1.32	0.67–2.60	0.42	1.15	0.74–1.80	0.54	2.37	1.11–5.05	**0.03**
Neurobehavioral												
Total anxiety	0.13	0.51–0.48	0.48	0.89	0.52–1.54	0.68	1.13	0.78–1.65	0.52	0.89	0.51–1.55	0.68
State anxiety	1.05	0.75–1.45	0.79	0.79	0.47–1.36	0.40	1.07	0.75–1.54	0.71	0.77	0.45–1.34	0.36
Trait anxiety	1.24	0.86–1.78	0.25	1.05	0.63–1.74	0.86	1.18	0.80–1.75	0.41	1.09	0.64–1.83	0.76
Geriatric depression	0.91	0.64–1.30	0.61	0.82	0.45–1.48	0.50	1.05	0.71–1.55	0.82	0.90	0.51–1.58	0.71
Cognitive domains^b^												
Global	0.95	0.69–1.31	0.74	0.91	0.58–1.43	0.69	1.11	0.78–1.58	0.56	0.70	0.43–1.16	0.17
Memory	0.93	0.55–1.59	0.80	1.09	0.42–2.82	0.87	0.77	0.43–1.38	0.38	0.61	0.23–1.64	0.33
Visuospatial	0.95	0.65–1.40	0.80	0.94	0.51–1.74	0.84	0.83	0.54–1.26	0.38	0.72	0.38–1.37	0.32
Working memory-executive	1.08	0.76–1.53	0.67	0.98	0.53–1.84	0.96	1.07	0.74–1.56	0.71	1.12	0.59–2.14	0.73
Attention-processing speed	0.85	0.57–1.27	0.44	0.83	0.42–1.61	0.57	0.79	0.51–1.23	0.30	0.66	0.32–1.34	0.25
Autonomic												
SCOPA-AUT	1.46	0.98–2.19	0.07	2.05	1.12–3.74	**0.02**	1.75	1.12–2.73	**0.01**	1.55	0.8–2.89	0.16
PD motor subtypes												
PIGD vs. TD	2.16	0.86–5.40	0.10	0.37	0.09–1.57	0.18	0.98	0.37–2.63	0.97	0.72	0.19–2.81	0.64
IND vs. TD	1.84	0.64–5.24	0.26	0.06	0.01–0.86	**0.04**	0.99	0.32–3.07	0.98	0.57	0.07–4.43	0.59

CI = Confidence Interval; NMS = non-motor symptoms; OR = Odds Ratio; IND = intermediate; PD = Parkinson disease; PIGD = postural instability and gait disturbance; TD = tremor dominant; UPDRS = Unified Parkinson’s Disease Rating Scale. Bold text indicates statistically significant (*p* < 0.05).

a Adjusted for age at baseline, race, education, disease duration, UPDRS III motor scores, and H&Y stage.

b The global domain included the Montreal cognitive assessment test. The memory domain included the immediate recall, delayed recall, and delayed recognition of the Hopkins verbal learning test–revised. The visuospatial domain included the Benton judgment of line orientation. The working memory–executive domain included the letter number sequencing and the semantic fluency–animal tests. The attention processing speed included the symbol digit modalities test.

### 3.4 Comparisons between self-perceived and examiner-rated speech severity

**Figure 1:**
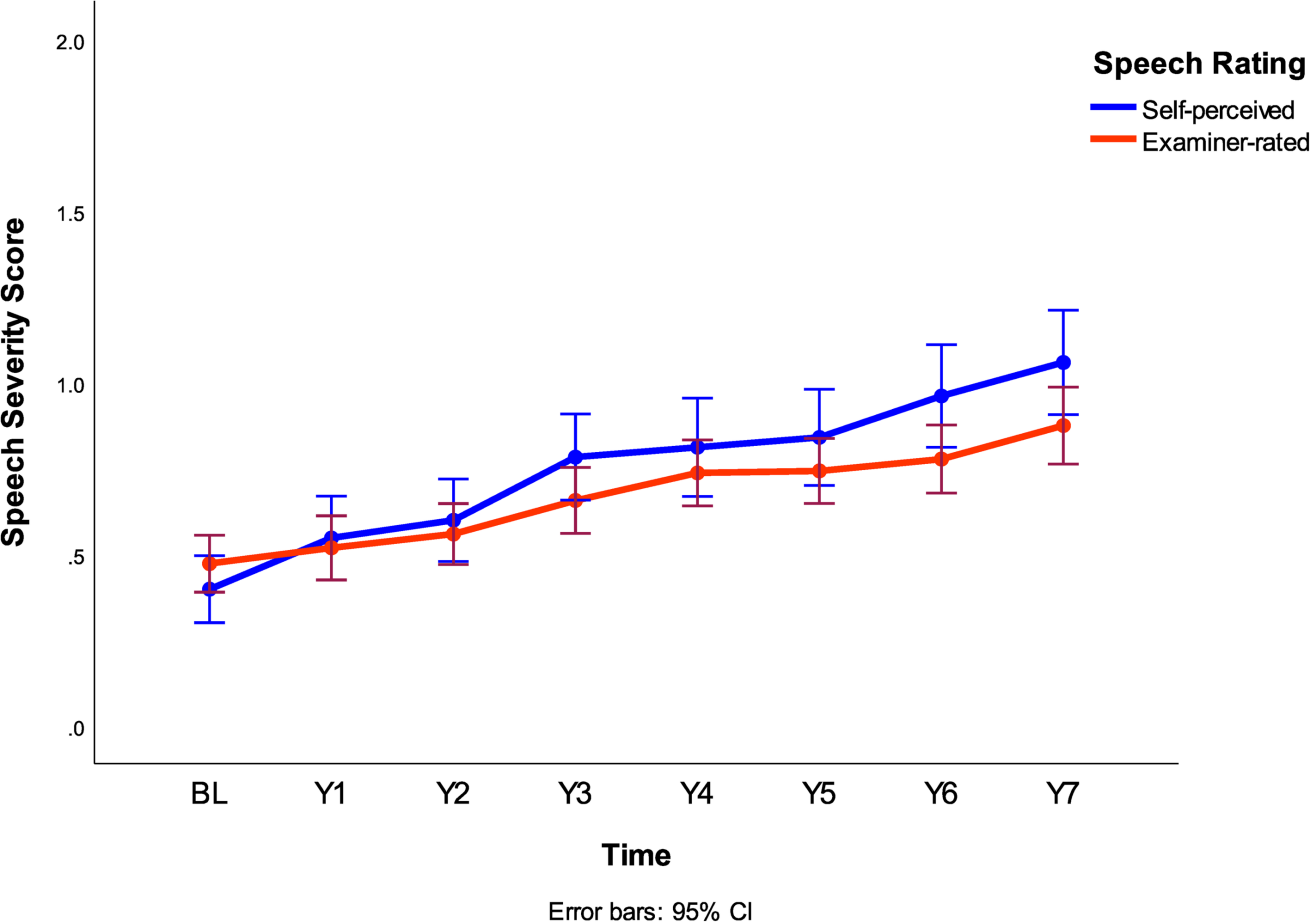
Self-perceived and examiner-rated speech severity ratings in Parkinson’s disease across a 7-year period from baseline. Error bars represent 95% confidence intervals.

Friedman’s repeated measures test showed a significant difference in speech severity across the seven visits for both self-perceived (χ^2^ = 140.77, *p* < 0.001) and examiner-rated ratings (χ^2^ = 98.77, *p* < 0.001). Figure 1 shows the progression of self-perceived and examiner-rated speech severity ratings at baseline and across a seven-year period. Self-perceived and examiner-rated speech impairment severity ratings were similar at baseline and years 1 to 5, but significantly higher self-perceived ratings at year six (*p* = 0.013) and seven (*p* = 0.013). However, after Bonferroni correction (*p* < 0.006) was applied to account for multiple comparisons, no statistically significant pairwise differences remained.

### 3.5 Sex-stratified speech severity trajectories across follow-up

**Figure 2:**
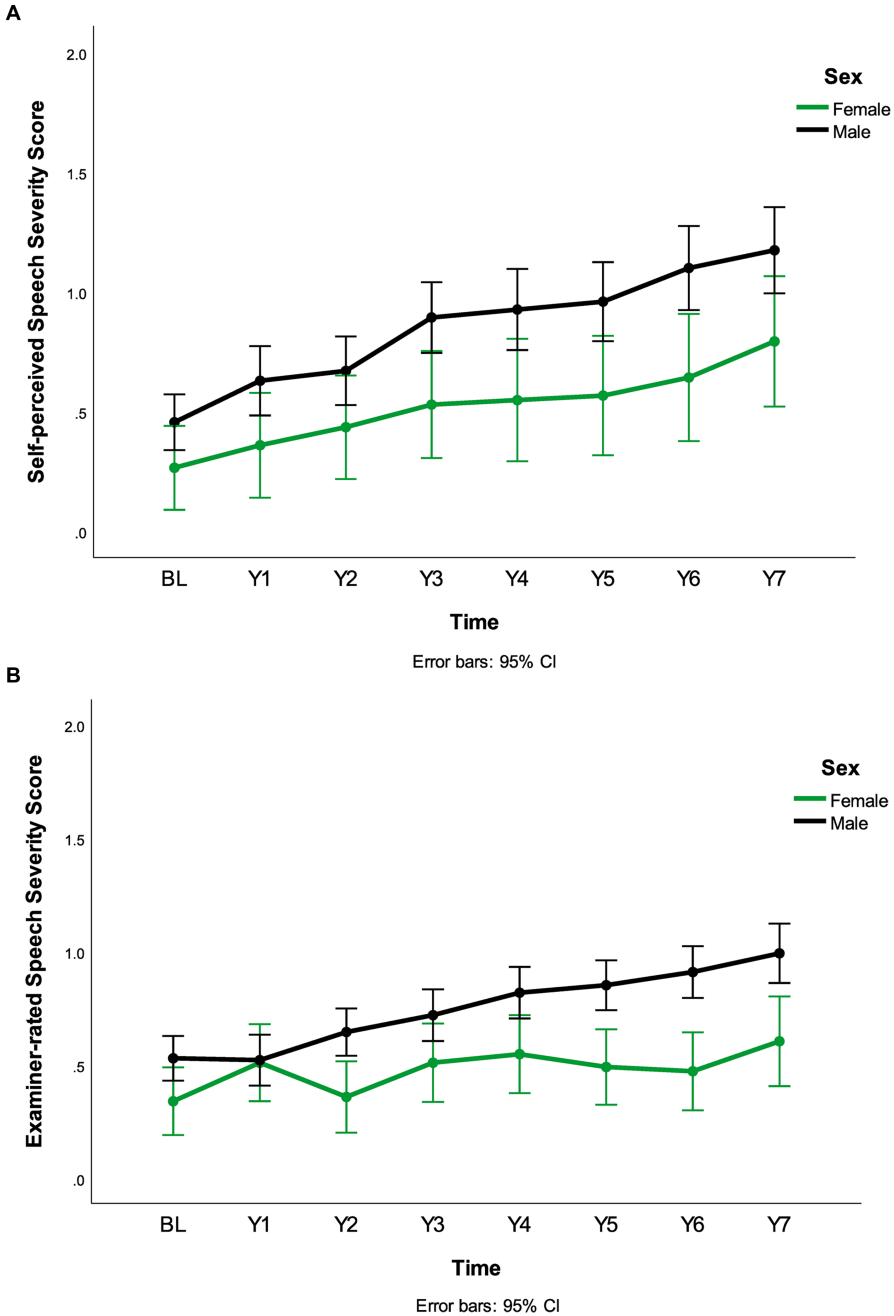
Sex-stratified self-perceived and examiner-rated speech severity ratings in Parkinson’s disease across a 7-year period from baseline. **(A)** Self-perceived speech severity ratings. **(B)** Examiner-rated speech severity ratings. Error bars represent 95% confidence intervals.

Analyses of sex-specific speech severity trajectories over the seven-year follow-up period revealed differential patterns for self-perceived and examiner-rated speech ratings (Figure 2). For self-perceived speech severity, similar longitudinal trajectories were observed between males and females (Wald χ^2^ = 1.74, *p-interaction* = 0.973), with both groups showing a comparable gradual increase in self-perceived speech severity across follow-up visits (Figure 2A). In contrast, a statistically significant sex difference was observed in examiner-rated speech severity trajectories over the follow-up period (Wald χ^2^ = 18.94, *p-interaction* = 0.008), with males showing a greater increase in speech severity over time compared to females (Figure 2B).

## 4 Discussion

This study examined the longitudinal progression of perceptual speech impairments in PWPD and the role of early NMS in relation to speech severity at year seven. Several baseline NMS, including RBD and autonomic dysfunction, were significantly associated with worse speech severity at year seven, with notable sex differences observed. Both self-perceived and examiner-rated speech severity progressively worsened over time in a parallel pattern, showing strong agreement between the two assessments.

In the present study, significant sex differences in self-perceived and examiner-rated speech severity ratings were observed among completers, with males having higher odds of worsening speech severity at year seven. While the neuropathological mechanisms underlying sex differences remain unclear, prior research has suggested that males have a higher prevalence of non-tremor dominant motor phenotype than females (20), which, has been associated with more severe speech impairments (21, 22). Although we did not observe an association between PD motor phenotype and speech impairment severity, prior studies have reported that speech impairments are more common in patients with an akinetic-rigid dominant motor subtype (3) and more pronounced in those with a postural instability and gait difficulty subtype (22), compared to patients with a tremor-dominant subtype. In our study, the tremor-dominant phenotype was the most common motor subtype in both males and females. Further research is needed better understand the interplay between sex and motor phenotype in predicting speech severity and progression in PD.

NMS, such as REM sleep behavior disorder, daytime sleepiness, and depression, often precede motor symptoms (23) and contribute significantly to disability (12). In the present study, greater baseline RBD and autonomic dysfunction symptoms were associated with worse self-perceived and examiner-rated speech severity at year seven. In contrast, higher baseline daytime sleepiness was associated with worse examiner-rated speech scores at year seven but was not related to self-perceived speech severity. Notably, these associations appeared to differ by sex. Higher RBD scores were associated with worse self-perceived speech ratings in males but not females, whereas in females, RBD symptoms were associated with worse examiner-rated speech severity. Additionally, greater autonomic dysfunction at baseline was linked to higher self-perceived speech severity ratings in females but higher examiner-rated severity in males.

The relationship between early NMS and PD progression remains complex. RBD and autonomic dysfunction have been increasingly recognized as markers of a more severe PD phenotype (24–26). Autonomic dysfunction, in particular, has been shown to be a stronger predictor of disease progression than RBD, and the co-occurrence of these symptoms has been associated with more severe motor and non-motor symptom profiles, possibly resulting from widespread *α*-synuclein pathology (25). In addition, recent evidence suggests that RBD is associated with more widespread and symmetric neurodegeneration involving both nigrostriatal and brainstem neurotransmitter systems (26). Our findings that baseline RBD and autonomic dysfunction were associated with worse speech impairments at year seven may therefore reflect the impact of widespread neurodegeneration on multiple neural systems involved in speech production (27). Moreover, previous studies have also found that PWPD with speech impairments at baseline exhibit significantly greater autonomic dysfunction, RBD symptoms, and daytime sleepiness (3). Finally, while depression can negatively impact speech acoustics and prosody, and is more prevalent in PWPD than in healthy controls (12), it was not significantly associated with speech ratings at baseline in our study and did not predict worsening speech severity over time. Future longitudinal studies are needed to elucidate the underlying mechanisms of these associations and to determine whether combinations of NMS can better identify individuals at risk for worse speech outcomes over time.

Self-awareness of speech deficits is important for timely identification of a need for speech therapy. In the present study, self-perceived speech impairment in PWPD worsened over a seven-year period, extending previous findings that indicate a mild but progressive decline in speech self-perception ratings among individuals newly diagnosed with PD (9). A similar pattern was observed in examiner-rated speech impairment, showing strong agreement between the two assessments. This suggests that PWPD maintain good insight into the overall severity of their speech deficits. Elucidating the ability of PWPD to accurately self-assess their speech skills has been the target of several studies, with mixed results. Contreras-Ruston et al. (28) found that PWPD, despite exhibiting reduced loudness, self-reported fewer voice symptoms than individuals with other voice disorders when assessed using a validated questionnaire. In contrast, another study found that individuals with early to mild stage PD who accurately rated their own speech samples reported lower articulation and speech intelligibility scores compared to healthy controls in supported tasks (29). Furthermore, PWPD has been shown to effectively self-identify speech impairment using a tailored screening tool (21). Differences in findings across studies may be attributable to the complex nature of PD-related symptoms (28), as well as variability in rating tasks (29) and instruments used for speech assessment.

In the current study, cognition at baseline did not predict self-perceived or examiner-rated speech impairment severity ratings over time. This lack of relationship between cognition and speech impairments contrasts with previous findings. For example, Polychronis et al. (3) observed that presence of observer-perceived speech impairment in *de novo* PWPD predicted cognitive impairment three years later. Similarly, Rektorová et al. (30) noted that impaired speech prosody in individuals diagnosed with mild to moderate PD predicted worsening cognition two years later. The lack of significant findings in the present study may be attributable to differences in study methodology.

Our study has several notable strengths including the use of longitudinal PD cohort data. The large sample size of newly diagnosed PD patients allowed for meaningful sex-specific analyses and between group comparisons. Furthermore, the assessment of a wide range of NMS assessed at baseline, using validated instruments, offered a unique opportunity for us to examine multiple NMS simultaneously in relation to speech progression in subsequent years. Nevertheless, this study has several limitations. First, objective neurophysiological assessments of NMS, such as polysomnography (PSG), the gold standard for the diagnosis of RBD, and autonomic reflex testing for autonomic dysfunction, were not available in the PPMI cohort. Future studies incorporating such objective assessments may provide a more precise characterization of these non-motor symptoms. Second, speech impairments were evaluated based on perceptual judgments using individual item rating scores from the MDS-UPDRS. Speech acoustic data, or other objective measures, were not available. While perceptual measures are important components when developing a profile of speech abilities, speech acoustic or other objectives measures are typically gathered to complement the subjective ratings. Third, in the present study, examiners are instructed to rate “modulation, diction of volume” (MDS-UPDRS 3.1). These terms are broad, and may be perceived differently by speakers, and inter-rater agreement on ratings is unknown. Indeed, inter-rater and intra-rater reliability in judgement of voice depends upon terms used (31), even among trained listeners. Forth, the prompt for examiner ratings of speech differs from the prompt provided to PWPD for self-rating of speech (MDS-UPDRS 2.1). The latter requires the PWPD to indicate whether or not they experienced a problem with speech over the past week. While both prompts elicit judgments of speech, due to differences in wording, caution must be taken when comparing ratings. Additionally, differences in interpretation of instructions for eliciting a speech sample may impact ratings (32). Fifth, medication status was not available for all participants at all visits. Consequently, we could not control for “on”/ “off” status in the analysis. However, previous studies have shown that the decline in speech motor function is only weakly correlated with motor changes and is largely unaffected by dopaminergic treatment (33). Finally, the PPMI cohort comprises predominantly white volunteers, which limits the generalizability of findings. However, the clinical characteristics of PPMI patients are typical of general patients with PD, and the PPMI investigators’ commitment to detailed clinical examinations ensures high-quality data collection.

## 5 Conclusion

Our findings suggest that certain NMS, particularly REM sleep behavior disorder and autonomic dysfunction, are associated with greater perceived speech impairments, with notable differences observed by sex. Both self-perceived and examiner-rated speech impairment severity worsened over time and remained largely consistent. Future studies should further explore factors influencing perceived speech decline to identify candidates who may benefit from early speech interventions aimed at preserving communication skills and quality of life.

## Data Availability

Publicly available datasets were analyzed in this study. This data can be found here: https://www.ppmi-info.org/access-data-specimens/download-data.

## Footnotes

1
http://www.ppmi-info.org/study-design/

2
www.ppmi-info.org/access-dataspecimens/download-data
