# Supplementary material for: Associations of non-motor symptoms with perceptual speech impairments in Parkinson’s disease
Source: Front Neurol. 2026 Jun 24;17:1827374. doi: 10.3389/fneur.2026.1827374 (PMC13341526; doi:10.3389/fneur.2026.1827374)
Supplement: Supplementary file 1 [file Table_1.DOCX]

**Supplementary Table S1. Non-motor Assessment Questionnaires**

| **Sleep Disorder Tests** |
| --- |
| Epworth Sleepiness Scale (ESS)  REM Sleep Behavior Disorder Screening Questionnaire (RBDSQ) |
| **Olfactory Test** |
| University of Pennsylvania Smell Identification Test (UPSIT) |
| **Neurobehavioral Tests** |
| State-Trait Anxiety Inventory for Adults (STAI)^a^  Questionnaire for Impulsive-Compulsive Disorders in Parkinson’s Disease (QUIP-Short)  Geriatric Depression Scale (Short Version) (GDS-15) |
| **Autonomic Tests** |
| Scales for Outcomes in Parkinson's Disease-Autonomic Questionnaire (SCOPA-AUT) |
| **Neuropsychological Tests and Cognitive Domains** |
| *Global*  Montreal Cognitive Assessment (MoCA)  *Memory*  Hopkins Verbal Learning Test – Revised (HVLT-R)^a^  *Visuospatial*  Benton Judgment of Line Orientation (JOLO) (15-item version)  *Working memory-executive*  Letter Number Sequencing (LNS)  Semantic Fluency (Animal, Vegetable, Fruit)^b^  *Attention-processing speed*  Symbol Digit Modalities Test (SDMT) |

^a^ Subcategories of STAI (trait vs. state anxiety) and HVLT-R (immediate vs. delayed recall vs. delayed recognition) were included in the analysis to allow for a more holistic examination of these possible symptoms.

^b^ Since established normalized scores exist only for animal fluency, we therefore did not include results on vegetable and fruit fluency in our analyses.
