# Supplementary material for: Associations of non-motor symptoms with perceptual speech impairments in Parkinson’s disease
Source: Front Neurol. 2026 Jun 24;17:1827374. doi: 10.3389/fneur.2026.1827374 (PMC13341526; doi:10.3389/fneur.2026.1827374)
Supplement: Supplementary file 2 [file Table_2.DOCX]

**Supplemental Table S2.** Associations between baseline non-motor symptoms and longitudinal changes in speech severity from baseline to year 7

|  | **Self-perceived Speech Severity Rating** | | **Examiner-rated Speech Severity Rating** | |
| --- | --- | --- | --- | --- |
|  | **β (95%CI)** | ***P* Value**^a^ | **β (95% CI)** | ***P* Value^a^** |
| **Sleep Disorder** |  |  |  |  |
| Epworth sleepiness scale | 0.04 (-0.11, 0.20) | 0.58 | 0.00 (-0.12, 0.13) | 0.99 |
| RBDSQ | 0.19 (0.04, 0.34) | 0.01 | 0.10 (-0.02, 0.22) | 0.09 |
| **Olfactory** |  |  |  |  |
| UPSIT | -0.08 (-0.26, 0.10) | 0.39 | 0.07 (-0.07, 0.22) | 0.31 |
| **Neurobehavioral** |  |  |  |  |
| Total anxiety | 0.05 (-0.10, 0.20) | 0.51 | 0.08 (-0.04, 0.20) | 0.20 |
| State anxiety | 0.03 (-0.12, 0.18) | 0.67 | 0.04 (-0.08, 0.16) | 0.49 |
| Trait anxiety | 0.06 (-0.09, 0.21) | 0.43 | 0.10 (-0.02, 0.23) | 0.09 |
| Geriatric depression | -0.07 (-0.22, 0.09) | 0.39 | 0.02 (-0.10, 0.15) | 0.69 |
| **Cognitive Domains**^b^ |  |  |  |  |
| Global | -0.07 (-0.20, 0.07) | 0.32 | -0.11 (-0.21, 0.00) | 0.05 |
| Memory | -0.11 (-0.34, 0.13) | 0.37 | -0.26 (-0.44, -0.08) | 0.01 |
| Visuospatial | -0.03 (-0.20, 0.13) | 0.71 | -0.04 (-0.17, 0.10) | 0.59 |
| Working Memory-Executive | 0.01 (-0.15, 0.17) | 0.90 | -0.00 (-0.13, 0.12) | 0.98 |
| Attention-Processing Speed | -0.04 (-0.22, 0.14) | 0.65 | -0.10 (-0.24, 0.04) | 0.14 |
| **Autonomic** |  |  |  |  |
| SCOPA-AUT | 0.10 (-0.06, 0.27) | 0.22 | 0.04 (-0.10, 0.17) | 0.60 |

Abbreviations: β = regression coefficient; CI = confidence interval

^a^ Adjusted for age at baseline, race, education, disease duration, UPDRS III motor scores, and H&Y stage.

^b^ The global domain included the Montreal Cognitive Assessment test. The memory domain included the immediate recall, delayed recall, and delayed recognition of the Hopkins Verbal Learning Test–Revised. The visuospatial domain included the Benton Judgment of Line Orientation. The working memory–executive domain included the Letter Number Sequencing and the Semantic Fluency–Animal tests. The attention processing speed included the Symbol Digit Modalities Test.
