# Supplementary material for: Associations of non-motor symptoms with perceptual speech impairments in Parkinson’s disease
Source: Front Neurol. 2026 Jun 24;17:1827374. doi: 10.3389/fneur.2026.1827374 (PMC13341526; doi:10.3389/fneur.2026.1827374)
Supplement: Supplementary file 3 [file Table_3.docx]

**Supplementary Table S3**. List of participating PPMI clinical sites.

| **Location** | **Organization** |
| --- | --- |
| Sun City, AZ | Banner Sun Health Research Institute |
| Phoenix, AZ | Barrow Neurological Institute |
| Houston, TX | Baylor College of Medicine |
| Boston, MA | Boston University Medical Center |
| Las Vegas, NV | Cleveland Clinic Lou Ruvo Center for Brain Health |
| Newcastle upon Tyne, England | Clinical Ageing Research Unit, Newcastle University |
| Atlanta, GA | Emory University School of Medicine |
| Barcelona, Spain | Hospital Clinic de Barcelona |
| Donostia-San Sebastian, Spain | Hospital Universitario Donostia |
| London, United Kingdom | Imperial College London |
| New Haven, CT | Institute for Neurodegenerative Disorders/XingImaging |
| Oxford, United Kingdom | John Radcliffe Hospital Oxford and Oxford University |
| Baltimore, MD | Johns Hopkins University |
| Los Angeles, CA | Keck School of Medicine of USC |
| Lagos, Nigeria | Lagos College of Medicine, University of Lagos |
| Boston, MA | Massachusetts General Hospital |
| Scottsdale, AZ | Mayo Clinic of Arizona |
| Innsbruck, Austria | Medical University Innsbruck |
| Montreal, Canada | Montreal Neurological Institute-Hospital |
| New York, NY | Mount Sinai Beth Israel |
| Athens, Greece | National and Kapodistrian University of Athens |
| Chicago, IL | Northwestern University |
| New York, NY | NYU Langone Health |
| Portland, OR | Oregon Health and Science University |
| Kassel, Germany | Paracelsus-Elena Klinik Kassel |
| Boca Raton, FL | Parkinson's Disease and Movement Disorders Center of Boca Raton |
| Marburg, Germany | Philipps-University of Marburg |
| London, United Kingdom | Queen Mary East London (QMUL) |
| Nijmegen, Netherlands | Radboud University |
| Tel Aviv, Israel | Tel Aviv Sourasky Medical Center |
| Cleveland, OH | The Cleveland Clinic |
| Ottawa, Canada | The Ottawa Hospital |
| Toronto, Canada | Toronto Western Hospital |
| Birmingham, AL | University of Alabama at Birmingham |
| La Jolla, CA | University of California, San Diego |
| San Francisco, CA | University of California, San Francisco |
| Cincinnati, OH | University of Cincinnati |
| Aurora, CO | University of Colorado Denver |
| Gainesville, FL | University of Florida |
| Kansas City, KS | University of Kansas Medical Center |
| Luebeck, Germany | University of Luebeck |
| Luxembourg, Luxembourg | University of Luxembourg |
| Ann Arbor, MI | University of Michigan |
| Philadelphia, PA | University of Pennsylvania |
| Pittsburgh, PA | University of Pittsburgh |
| Rochester, NY | University of Rochester |
| Salerno, Italy | University of Salerno |
| Tampa, FL | University of South Florida |
| Tübingen, Germany | University of Tuebingen |
| Seattle, WA | VA Puget Sound Health Care System |
